# Melanoma in the Vulva of a 71-Year-Old Patient: A Case Report

**DOI:** 10.7759/cureus.32698

**Published:** 2022-12-19

**Authors:** Yakubmiyer Musheyev, Michail Fazylov, Chiya Abramowitz, Teddy A Ikhuoriah, Peter Rogu, Maria Levada

**Affiliations:** 1 Medicine, New York Institute of Technology College of Osteopathic Medicine, Old Westbury, USA; 2 Obstetrics and Gynaecology, New York Institute of Technology College of Osteopathic Medicine, Old Westbury, USA

**Keywords:** immunohistochemistry, treatment, mucosal malignant melanoma, vulva cancer, melanoma

## Abstract

Mucosal melanomas (MM) are a rare type of melanomas commonly found in the vulvovaginal, anorectal, and respiratory tract. In this case report, a 71-year-old female presented to her OB/GYN clinic with dark raised mass on her right labial region adjacent to the perineum. Past medical and surgical history of note included third-degree uterine prolapse, senile vaginitis, fibrocystic changes of the breasts bilaterally, hypothyroidism, hypertension, as well as a past hysterectomy and anterior colporrhaphy. Upon further workup, the 2.7 x 1.8 x 2 cm polyploid mass was biopsied and was found to be consistent with malignant melanoma. The patient then underwent a wide local excision confirming that the lesion was a nodular vulvar melanoma with superficial ulcerations and lymphovascular invasion of the vulvar region. Post-wide local incisions were found to be healed well after the procedure and the patient was referred to a gynecological oncologist for continuous monitoring. The purpose of this case report is to bring awareness of melanomas arising in atypical regions. While MMs are rare in comparison to cutaneous melanomas (CM), the prognosis can be poor if not caught early.

## Introduction

Melanoma is a malignancy of melanocytes that arises from the basal layer of the epidermal skin cells due to uncontrolled proliferation [1]. Cutaneous melanoma (CM) accounts for 1.7% of global cancer diagnoses and is the fifth most common cancer in the United States with 5.2% of all new cancer cases as of 2022 [1-3]. Mucosal melanomas (MM) account for 1.4% of all melanomas and are rare in comparison but have a poorer prognosis [4,5]. In addition, MM-defined precursor lesions have not been identified at this time [5]. On the genetic level, CM patients are more prone to single nucleotide mutations, fewer chromosomal variations, and increased UV light mutations when compared with MM groups [5-7]. Furthermore, BRAF and NRAS mutations are more common in CM patients while PTEN, KIT, and CDKN2A mutations have a higher likelihood within MM-sampled patients [5,7].

MMs are able to appear on any mucosal surface but tend to emerge in three common areas; vulvovaginal, the anorectum, and the respiratory tract [6]. The signs and symptoms of MM vary based on its origin. The most common symptoms of MM of the respiratory tract are nasal obstructions, lesions, and epistaxis with more aggressive forms showing facial pain and distortion [8]. Within the vulvovaginal region, symptoms include bleeding, itchiness, and bulging masses [8]. Gastrointestinal MM includes masses in the oral cavity that may be pigmented and elevated [8]. Diagnosing primary MM is often difficult due to its hidden locations, lack of early symptoms, and possible metastases [5,8]. Moreover, lack of pigment and junctional changes make the diagnosis even harder with similarities of lymphoma and angiosarcoma [8]. Immunohistochemical staining for S-100, tyrosinase, and HMB-45 aid in a malignant melanocytic diagnosis [8]. Treatment for CM and MM patients is often patient-specific with an emphasis on surgical excision while other therapies are currently being pursued [8].

## Case presentation

A 71-year-old gravida 2 para 2 (G2P2) Caucasian female who had been post-menopausal for over 20 years, presented to her obstetrics and gynecology (OB/GYN) physician with a chief complaint of an irritating growth on her right labia for the previous two months. Physical examination revealed a dark raised mass on the lower right labia adjacent to the perineum, with no significant vulvar atrophy. There was no urethral discharge. The vagina was noted to have atrophy and inflammation but was not painful.

There were no suspicions of malignancy at the time of presentation; previous cervical and breast cancer screenings were all negative. Signs of melanoma or any characteristic skin changes associated with melanoma were not found on any other region of the body at the time of presentation.

Her past history included a complete transvaginal hysterectomy with anterior and posterior colporrhaphy for a third-degree uterine prolapse with cystocele and rectocele 30 years prior. She also had a past history of fibrocystic changes of the breast bilaterally, left breast nodular density 15 years prior (which was being followed up by a breast specialist), menopausal syndrome, and senile vaginitis. Her last pap smear was three years prior and was normal. Her non-gynecological history included hypertension and hypothyroidism for which she was taking medication, a small hiatal hernia, degenerative changes in the lumbar spine, pyelonephritis, and hepatitis. She had no family history of breast, gynecological, or skin cancers.

The main differential diagnosis for a vulvar mass in this age group includes benign nevus, dysplastic nevus, squamous cell carcinoma of the vulva, and melanoma.

**Figure 1:**
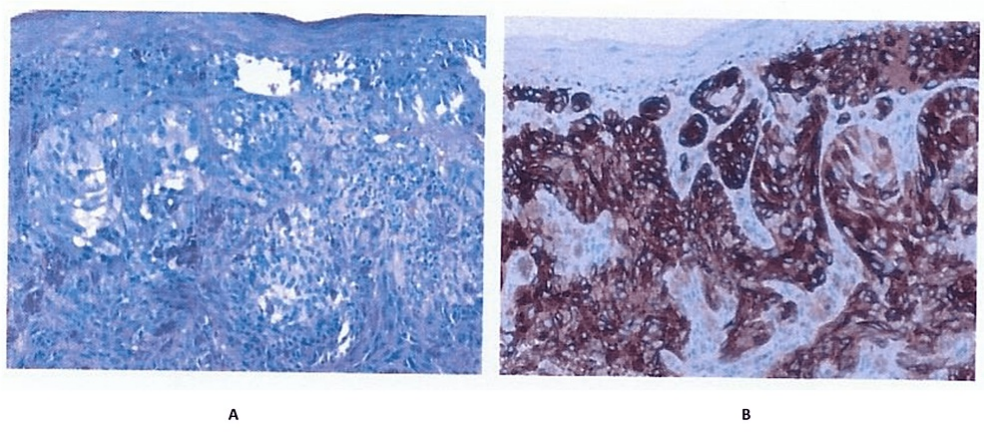
H&E stain (A) and Melan-A immunohistochemical stain (B) of office biopsy

The patient underwent an office biopsy with H&E and Melan-A staining (Figure 1). Of note, the Melan-A immunohistochemically staining (Figure 1) panel B showed brown staining that positively confirmed melanoma. The biopsy report stated there was a malignant melanoma with no definite presence of ulceration. Lymphovascular or perineural invasion was not demonstrated. There were also focal changes suggestive of regression phenomenon. The report also noted that there was one mitosis per square millimeter and that the inflammatory response was minimal. There was also no evidence of a precursor melanocytic lesion. Complete excision of the lesion with appropriate margins of the normal surrounding tissue was warranted/recommended.

The patient was further referred for a pre- and post-contrast abdominal CT scan to see if there was a possibility of any abnormalities that could be related to the melanoma. The CT scan was negative and revealed no retroperitoneal lymphadenopathy.

Approximately one month after the initial diagnostic biopsy, the patient underwent a wide local excision via an ambulatory in-hospital procedure. The gross findings of the tumor showed a 2.7 x 1.8 x 2 cm polyploid piece of black and tan tissue having a smooth surface. The tumor had a Breslow thickness of 4.3 mm. The accessory finding of the tumor included ulceration, a mitotic rate of 9 mitoses/mm^2^, and lymphovascular invasion. Neurotropism, tumor-infiltrating lymphocytes, and tumor regression were not identified.

The surgical pathology report confirmed a right vulvar mass with nodular melanoma with superficial ulceration, and significant lymphovascular invasion in the right vulvar region. However, the margins were negative for invasive or in-situ melanoma. The hospital pathologist did an addended immunohistochemical study for BRAF V600E and it was negative.

The patient presented 1-week post-wide local excision for a post-op check. The incision was healing very well. A pelvic examination revealed no abnormalities, the vagina had no tenderness, erythema, vesicles, lesions, or discharge. A pelvic examination revealed no abnormalities. At this time, the patient was referred to a gynecologic oncologist where approximately 1-month post-operation, the patient underwent a biopsy for reevaluation. This biopsy revealed a melanoma in the right vulva, measuring at least 0.83 mm in thickness. Immunological testing of the specimen was positive for p16, HMB45, and Melan-A supporting the diagnosis of melanoma.

The patient was scheduled for a follow-up surgical excision by the gynecological oncologist.

## Discussion

Melanoma is a malignancy of multifactorial etiologies which includes genetic mutation and ultraviolet radiation [5,7]. The genetic mutations are often related to BRAF, NRAS, PTEN, KIT, and CDKN2A genes, though other genes have also been implicated [5,7,9]. Melanoma is more common in the elderly population but can also be seen in younger populations [9]. After age 50, it seems to be more common in males but before age 50 it is more common in females [9]. Other risk factors include dysplastic nevi (atypical mole), congenital melanocytic nevi, xeroderma pigmentosum, whites with red or blond hair, first-degree family history of melanoma, and decreased or weakened immunity [9].

Once a malignant melanoma is diagnosed and graded, an appropriate treatment plan for the individual patient must be prepared. Currently, depending on the stage, there are numerous treatment options available for such cancers ranging from surgical excision, photodynamic therapy, chemotherapy, immunotherapy, targeted drug therapy, or a combination of approaches [10]. The goal of all these is to maximize the benefit of treatment and minimize adverse effects experienced by the patient. For most melanomas, surgery is the primary treatment of choice [11]. A review of melanoma treatment outcomes has shown that the surgical excision of these tumors improves survivorship regardless of their location [12]. There is a greater efficacy associated with solitary lesions, much like in this case, as there is difficulty in excising metastatic tumors at distant sites [13]. With this in mind, other adjunct therapies can be incorporated to further improve survivorship. Most commonly, the treatments chosen are immunotherapy with interferon or ipilimumab [11]. Interferon α-2b is a signaling molecule that activates T-cells to promote anti-tumor activity [14]. In melanoma specifically, interferon α-2b stimulates major histocompatibility complex class I in the target cells and immune cells which leads to a dose-dependent apoptotic effect [15]. On the other hand, ipilimumab is an anti-immune checkpoints T-lymphocyte-associated protein 4 (CTLA-4) antibody that promotes anti-tumor activity [16]. CTLA-4 is a checkpoint inhibitor in the activation of T-cells and regulates immune response: this disinhibition by ipilimumab allows for the proliferation of T-cells [17]. While surgery with supplemental immunotherapy is the typical route of treatment, other previously mentioned options can be considered depending on the needs of the patient.

Melanoma of the vulva is rare, but cases are well documented. One case looked at a 70-year-old woman from India diagnosed with this malignancy [18]. She presented with non-tender swelling of the labial region for 2 months. She also complained of white, non-foul smelling, and not blood-stained discharge of the vagina over this same time period. Medical and surgical history were non-significant and on examination, her vitals were within normal limits. The mass was confirmed as melanoma via histology and was subsequently treated through surgical excision. This case presented a tumor of similar severity and location to ours. Both patients were of similar age, and both were treated surgically.

Traditionally, malignant melanoma is thought of as a disease of solar exposed areas. UV radiation-exposed areas such as the lower extremities in women and the trunk in men are most commonly affected [6]. Cases such as ours challenge this ideology and show that the disease may find its way even into the most sun-shielded parts of the body. While uncommon in the vulva, melanoma is deadly, so proper diagnosis and treatment are critical. We share this case with the aim of showing that even though the presentation of this disease is rare, careful consideration must be given to its possibility as its prognosis is poor if overlooked.

## Conclusions

Ultimately, in this case report, we presented a 71-year-old female with a chief complaint of an irritating growth on her right labia for the previous two months. Upon further workup of the patient, it was determined that the patient had a nodular melanoma in the vulva. Because of its location, it is classified as a MM. MMs are rare which makes this case noteworthy.
